# Draft genome sequence of the meloxicam-degrading *Gordonia alkanivorans* IEGM 1277 strain

**DOI:** 10.1128/mra.01071-25

**Published:** 2025-11-18

**Authors:** Semyon M. Tyan, Irina B. Ivshina

**Affiliations:** 1 Perm Federal Research Center of the Ural Branch of the Russian Academy of Sciences 307499, Perm, Russia; 2 Perm State University 64949, Perm, Russia; University of Southern California, Los Angeles, California, USA

**Keywords:** *Gordonia*, NSAIDs, meloxicam, biodegradation, draft genome sequence

## Abstract

We report the draft genome sequence of *Gordonia alkanivorans* IEGM 1277, an actinomycete strain exhibiting a pronounced ability to degrade the pharmaceutical pollutant meloxicam—an emerging environmental contaminant. Utilizing the acquired sequence, identify candidate genes that encode enzymes implicated in the degradation of meloxicam.

## ANNOUNCEMENT

Meloxicam is a commonly used non-steroidal anti-inflammatory drug in human and veterinary medicine and a pharmaceutical environmental contaminant (1, 2). It has moderate toxicity (LD_50_ for rats: 83.5 mg/kg) and notable lipophilicity (log *K*_ow_ 3.43), facilitating bioaccumulation, especially in aquatic organisms (3, 4). Meloxicam enters the environment primarily via wastewater due to inefficient removal in treatment plants and is detectable in their effluents (5, 6). Documented adverse effects include toxicity to fish, birds, and mammals (7–9).

Bioremediation using stress-resistant actinomycetes offers an eco-friendly and cost-effective approach for detoxifying xenobiotics (10). These microorganisms resist pharmaceutical pollutants and sustain hydrocarbon oxidation in the presence of trace metals, enabling effective remediation of complex contamination (11). The *Gordonia alkanivorans* strain IEGM 1277, isolated from oil-polluted waste in 2014 using the enrichment culture method, is deposited in the Regional Specialized Collection of Alkanotrophic Microorganisms (acronym IEGM, WFCC #285, http://iegmcol.ru/strains/gordonia/alkanivorans/g_alkanivorans1277.html). This strain completely degrades 10 mg/L meloxicam in mineral medium within 14 days, producing less (eco)toxic metabolites 5′-hydroxymethylmeloxicam and 5′-carboxymeloxicam (12).

DNA was isolated from *G. alkanivorans* IEGM 1277 cells grown in LB broth at 28°C and 160 rpm for 28 h using the MagMAX DNA Multi-Sample Ultra 2.0 kit and KingFisher Flex automation (Thermo Fisher Scientific), following the manufacturer’s protocol. Over 500 ng of DNA was obtained; 100 ng was used for sequencing. Genome sequencing was performed using NGS on the Illumina DNA Prep protocol. In total, 43,443,884 reads were obtained. Demultiplexing was performed with Illumina bcl2fastq (version 2.20). Adapter trimming used Skewer (version 0.2.2), and read quality was assessed via FastQC (version 0.11.5-cegat) (13). Quality trimming of the reads has not been performed. More than 95% reads had a Phred score ≥ 30. No reads were removed after the FastQC quality assessment. Reads were assembled *de novo* using SPAdes (version 3.14.1) (14), and assembly quality was evaluated with QUAST (version 5.2.0) (15). The annotation of coding sequences was performed using PGAP 5.3 (annotation method “best-placed reference protein set” GeneMarkS-2+) (16). The assembly consisted of 143 scaffolds and 149 contigs with a total sequence length of 5,116,883 bp, an *N*_50_ value of 149,232 bp, a GC content of 67.5%, and coverage of 672×. For taxonomic classification, average nucleotide identity was calculated using the online tool https://www.ezbiocloud.net/tools/ani (17). Digital DNA-DNA hybridization (dDDH) was calculated using GGDC (Genome-to-Genome Distance Calculator 4.0, https://ggdc.dsmz.de/ggdc.php#) (18, 19). The highest OrthoANIu (98.09%) and dDDH (81.2–86.4%) values were between the IEGM 1277 strain and type *G. alkanivorans* (NCBI: txid84096). Default parameters were used for all software unless otherwise specified.

The genome of *G. alkanivorans* IEGM 1277 contains 4,707 coding sequences, including 4,591 protein-coding genes, 4 rRNAs (5S, 16S, and 23S), and 48 tRNAs. Notably, it encodes 20 monooxygenases, 15 dioxygenases, 4 hydroxylases, 8 peroxidases, and 167 dehydrogenases. Among oxygenases, genes for 14 cytochrome P450 enzymes, 2 catechol 1,2-dioxygenases (EC 1.13.11.1), 1 alcohol dehydrogenase (EC 1.1.1.1), 2 nitronate monooxygenases (EC 1.13.12.16), and 1 homogentisate 1,2-dioxygenase (EC 1.13.11.5) were identified. These enzymes are likely involved in the biodegradation and metabolic pathway of meloxicam.

## Data Availability

This Whole-Genome Shotgun project has been deposited in GenBank under the accession number NZ_JASIRQ000000000.1 and the genome assembly number GCF_030063815.1. Raw sequence reads were deposited in the Sequence Read Archive under BioSample accession number SRR35263698, BioSample accession number SAMN35083989, and BioProject accession number PRJNA783162.
